# A novel single-tier serologic test to diagnose all stages of Lyme disease

**DOI:** 10.1128/jcm.00483-25

**Published:** 2025-08-20

**Authors:** Andrew E. Levin, Gary P. Wormser, Elizabeth J. Horn, Nadezhda Karaseva, Drew Miller, Hunter Kellogg

**Affiliations:** 1 Kephera Diagnostics, LLCFramingham, Massachusetts, USA; 2 Division of Infectious Diseases, Department of Medicine, New York Medical College 8137, Valhalla, New York, USA; 3 Lyme Disease BiobankPortland, Oregon, USA; Mayo Clinic Minnesota, Rochester, Minnesota, USA

**Keywords:** *Borrelia burgdorferi*, Lyme disease, serology, two-tier serology, *diagnosis*

## Abstract

Lyme disease, a bacterial zoonosis, is the most commonly reported vector-borne disease in the United States. Laboratory diagnosis has relied on a two-tier serologic approach, originally comprising an ELISA, or another first-tier assay, followed by separate IgG and IgM immunoblots to confirm a positive first-tier result. This standard two-tier testing (STTT) approach provides high specificity, but at the cost of low sensitivity in early Lyme disease. Recent studies have shown that a modified two-tier (MTTT) testing approach, in which a second ELISA replaces the immunoblot, can provide an increase in test sensitivity without a loss of specificity. Nevertheless, neither STTT nor MTTT is considered sensitive enough for diagnosing patients with erythema migrans, the most common clinical manifestation of early Lyme disease. We have developed a novel ELISA methodology termed “Hybrid Lyme ELISA” for single-tier Lyme antibody detection, which relies on the simultaneous binding of individual antibody molecules to the *Borrelia burgdorferi* surface protein VlsE and to the C6 peptide derived from it. This dual binding requirement builds exceptionally high specificity into the assay, eliminating the majority of non-specific antibody interactions. In this study, the single-tier Hybrid Lyme ELISA was shown to provide greater sensitivity, but with equivalent specificity, to both STTT and MTTT. In addition, given the >90% sensitivity of the Hybrid Lyme ELISA in patients with erythema migrans, this assay may not only transform serologic testing from two-step to single-step testing, but may also provide a means for the first time to diagnose patients with erythema migrans.

**IMPORTANCE:**

The diagnosis of Lyme disease, a tick-borne spirochetal infection caused by *Borrelia burgdorferi* sensu lato, is subject to two major limitations: the need for a two-tier serologic testing algorithm to provide adequate specificity, and the low sensitivity of this algorithm in practice for detection of early Lyme disease manifesting with the erythema migrans skin lesion, the most common clinical manifestation. This study presents the first description of a new assay, the Hybrid Lyme ELISA, which demonstrates sensitivity high enough to potentially diagnose over 90% of patients with erythema migrans, and specificity high enough to preclude the need for a second-tier test. These test characteristics suggest the potential for the Hybrid Lyme ELISA to be the first single-tier serologic test suitable for laboratory diagnosis of all stages of Lyme disease.

## INTRODUCTION

Lyme disease, a bacterial zoonosis principally caused by *Borrelia burgdorferi* sensu stricto in the United States, is the most common vector-borne disease reported in the country (1).

The earliest and most common clinical manifestation of Lyme disease is the skin lesion erythema migrans (EM). Currently, the diagnosis of EM must be made clinically, as currently recommended serologic testing approaches are not considered sensitive enough to be recommended for diagnosis of patients at this early stage of Lyme disease (2–5).

For non-EM disease manifestations, diagnosis presently relies on two-tier serologic testing that has been recommended by the CDC since 1994 (6). In the original standard two-tier testing (STTT) approach, serum specimens are tested first by an ELISA (or an immunofluorescence assay), and those found positive or indeterminate are subsequently tested by separate IgG and IgM immunoblot assays.

Unfortunately, the sensitivity of the STTT method may be as low as 30%–40% in early Lyme disease patients with EM, principally due to the relatively insensitive immunoblot step, which leads to false-negative results (3, 7–9). Removing the requirement for immunoblot confirmation would potentially double the sensitivity of serologic detection for early-stage Lyme disease (10), which would be a major benefit clinically if it could be achieved without reducing specificity. Indeed, a succession of studies showed that replacement of the immunoblot with a second ELISA in a serial “modified two-tier” (MTTT) algorithm resulted in higher sensitivity for patients with EM (but, however, still typically under 80%) (11–13), without a significant reduction in specificity (9, 14–16). While the MTTT has made the testing process more efficient and accessible, it has not changed the basic requirement for two separate serologic tests performed sequentially.

Our objective was to develop an ELISA that, as a single-tier assay, would offer sensitivity and specificity comparable to those of both the STTT and the MTTT algorithms. The assay that we have developed utilizes the unanticipated finding that a single multivalent antibody could simultaneously bind to both the VlsE protein and the C6 peptide. In this study, we compared the performance of this hybrid C6/VlsE ELISA (Hybrid Lyme ELISA) with that of both STTT and MTTT assays using panels of well-characterized Lyme disease patient and control sera.

## MATERIALS AND METHODS

### Serum samples

Twenty-five serum samples previously obtained from New York Medical College from patients with physician-documented EM, of which 22 were ELISA-positive (but second-tier testing was not performed), were included in this study. Twenty-seven sera were obtained from the Lyme Disease Biobank, including 21 from patients with physician-documented EM that were positive by STTT (17), and six additional EM sera from 3 patients from whom paired acute and convalescent sera were obtained. Eight sera from patients with symptoms of extracutaneous Lyme disease, which were positive by STTT, were also obtained previously from New York Medical College. Twenty-nine sera were obtained from commercial suppliers, all of which were positive by STTT but without information on the stage of Lyme disease; however, 25 of these 29 sera yielded an average of 16 bands on the IgG immunoblot, suggesting that they originated from patients with late-stage Lyme disease. In addition, the R67 Research Panel I was obtained from the CDC, which included four acute EM sera that were negative by STTT, four convalescent EM sera that were positive by STTT, and four sera from patients with extracutaneous manifestations of Lyme disease (two with neurologic Lyme disease and two with Lyme arthritis) that were also positive by STTT.

Control sera included 500 sera from blood donors from non-endemic regions in the United States, 64 sera from blood donors from endemic regions, and 57 sera from patients with other disease conditions. The numbers and categories of the sera tested in the present study are summarized in Table 1.

**TABLE 1:** Sources and characteristics of serum samples used in this study^*a*^

Panel	Acute EM, ELISA (1st tier) negative	Acute EM ELISA (1st tier) positive	Acute EM, STTT positive	Convalescent EM, STTT positive	STTT positive including extracutaneous*	Blood donor controls (endemic)	Blood donor controls (non-endemic)	Other disease conditions
CDC R67-I	4	–^*b*^	–	4	4*	4	4	12
NYMC	3	22	–	–	8*	–	–	–
LDB	–	–	21	–	6	60	–	–
Commercial	–	–	–	–	29	–	496	45
Total	7	22	21	4	47	64	500	57
	Total Lyme disease sera: *n* = 101					Total control sera: *n* = 621		

^*a*^ CDC R67-1, Research Panel I from the Centers for Disease Control and Prevention; NYMC, New York Medical College; LDB, Lyme Disease Biobank. Among STTT-positive sera, those from patients with known extracutaneous symptoms are marked with an asterisk.

^*b*^ "–" denotes no samples from that source in the category.

### Hybrid Lyme ELISA

**Fig 1:**
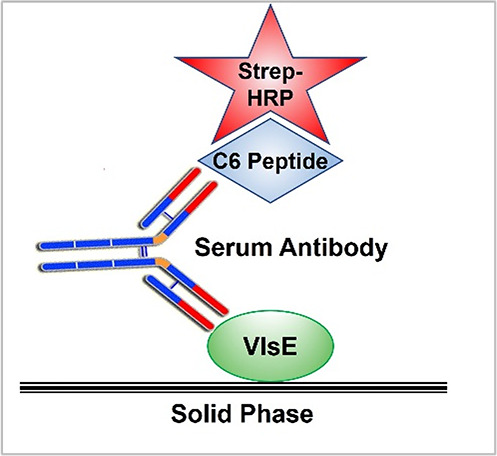
Hybrid Lyme ELISA schematic. Specific antibodies are detected by simultaneous binding to VlsE immobilized on solid phase and to biotinylated C6 peptide–streptavidin–HRP (horseradish peroxidase) conjugate.

The Hybrid Lyme ELISA kit consisted of a microplate coated with recombinant VlsE (GenScript, Piscataway, NJ) and a soluble, ready-to-use conjugate of C6 peptide (LifeTein, Somerset, NJ) with horseradish peroxidase (HRP), along with sample dilution and wash buffers, stop solution, and tetramethylbenzidine-containing HRP substrate solution. The assay principle is shown in Fig. 1.

**Fig 2:**
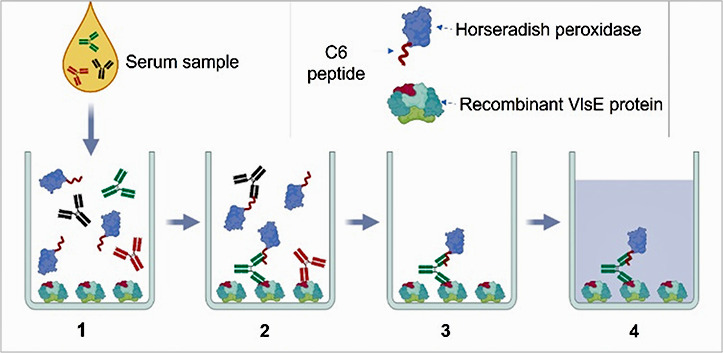
Hybrid Lyme ELISA Principle. (**1**) A serum or plasma sample is added to a microplate well coated with VlsE protein. (**2**) C6 peptide–HRP (horseradish peroxidase) conjugate is added to the same well immediately after the sample. Serum antibodies that recognize both C6 peptide and the homologous epitope in the VlsE protein bind both antigens simultaneously, thereby linking the VlsE–antibody–C6-HRP complex to the well. (**3**) Unbound antibodies and antibodies bound to either C6 peptide or VlsE, but not both, are removed by a wash step. (**4**) Bound HRP is then detected by the addition of HRP substrate.

The assay protocol comprised four main steps (Fig. 2). The serum sample (12 µL, neat) and C6 peptide–HRP conjugate solution (38 µL) were added simultaneously to the ELISA well, incubated for 60 min, and unbound material was removed by four buffer washes. The tetramethylbenzidine substrate (50 µL) was then added, incubated for 20 min, and the reaction quenched by the addition of 50 µL of stop solution. Absorbance was read using a Molecular Devices (SpectroMax 340) microplate reader at 450 nm.

**Fig 3:**
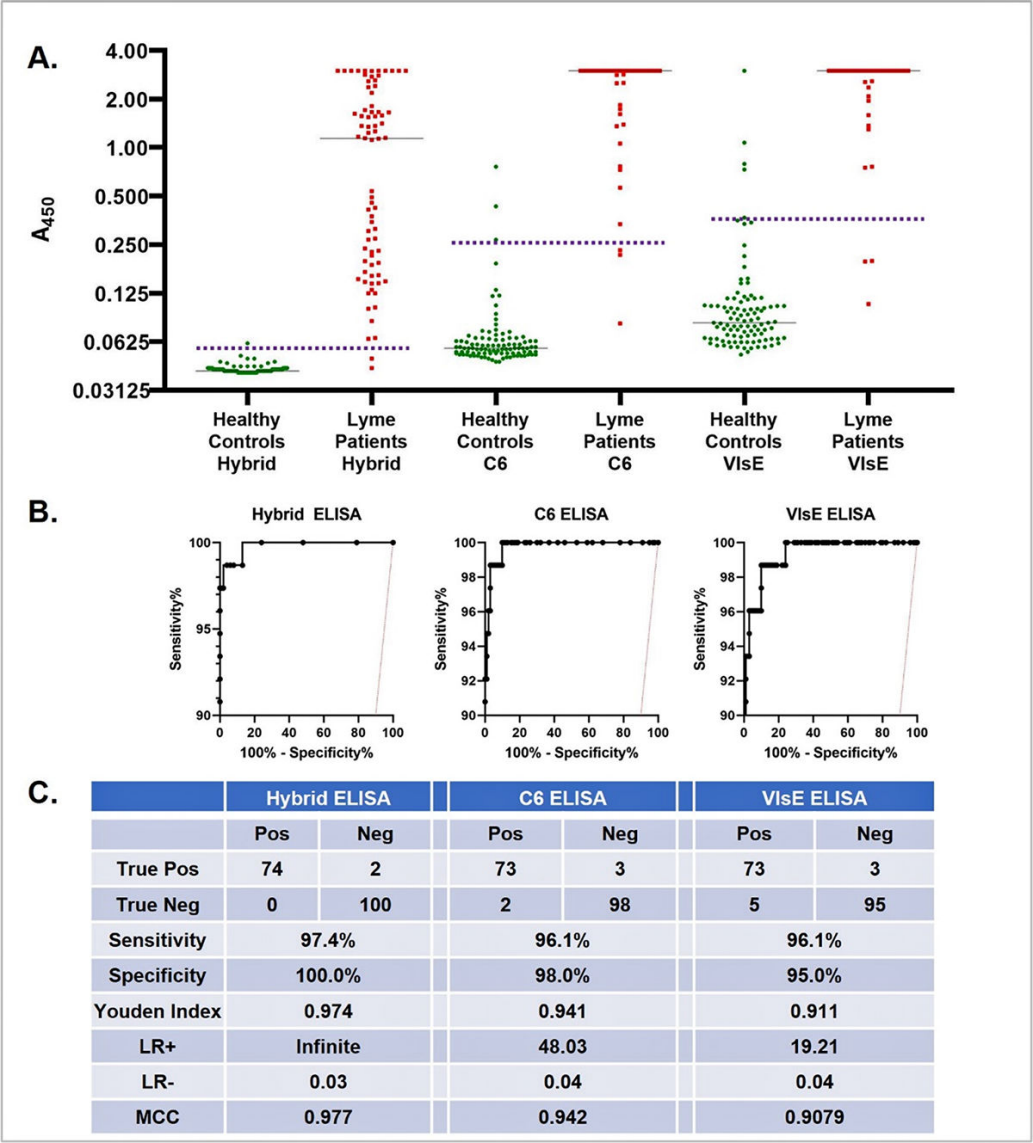
(**A**) Absorbance distribution. (**B**) ROC curves. (**C**) Sensitivity and specificity for 76 Lyme disease sera comprising 30 sera from patients presenting with EM, including both seropositive and seronegative sera, and 46 sera from Lyme disease patients with later stage symptoms, of which 45 were STTT-positive, and 100 blood donor sera tested on Hybrid Lyme ELISA, C6 ELISA, and VlsE ELISA. Dotted purple line indicates cutoff for each assay. Youden index = (sensitivity + specificity − 1) (18); LR^+^ (positive likelihood ratio) = sensitivity / (1 − specificity); LR^−^ (negative likelihood ratio) = (1 − sensitivity) / specificity); MCC (Matthews Correlation Coefficient) = [(TP × TN) − (FP × FN)] / SQRT[(TP + FP)·(TP + FN)·(TN + FP)·(TN + FN)], where TP = true positives, TN = true negatives, FP = false positives, FN = false negatives.

The cutoff for the Hybrid Lyme ELISA was derived from an ROC analysis of 76 two-tier positive Lyme disease sera and 100 blood donor sera (Fig. 3), except that six of the 76 Lyme disease sera shown replaced a similar number in the ROC analysis that were excluded due to lack of clinical characterization. A cutoff of 0.08 absorbance units yielded 96% sensitivity and 100% specificity on this ROC data set. We determined empirically that a cutoff value yielding similar sensitivity and specificity values could be reproducibly generated by adding the fixed value of 0.04, determined by averaging nine independent assay runs, to the absorbance of the negative control serum. That formula was then used to analyze the additional ELISA data presented in the manuscript. To interpret the result, the absorbance reading was divided by the cutoff to yield a Lyme index value (LI). Index values < 0.9 were interpreted as negative, 0.9 ≤ index value ≤ 1.1 as equivocal, and >1.1 as positive. Seventy of the positive and all 100 negative samples used in the study that led to the cutoff formula were included in the larger sample sets evaluated in Tables 4 and 5, but not in Table 3.

### C6 and VlsE ELISAs

Microplates were coated with either 1 µg/mL of streptavidin followed by biotinylated C6 peptide or with 2 µg/mL of recombinant VlsE protein and 8 µg/mL of bovine serum albumin to generate the respective ELISAs in conventional indirect format. The assay procedure consisted of a 1-h incubation with serum samples diluted 1:100 in 50 mM Tris buffer containing 0.1% casein, 150 mM NaCl, and 0.1% Tween 20, followed by three washes with PBS with 0.05% Tween 20. Plates were then incubated with goat anti-human IgG–horseradish peroxidase conjugate (Jackson Immunoresearch) at 1:20,000 dilution, followed by three more washes. Bound antibodies were revealed using tetramethylbenzidine (TMB, Moss). Absorbance was read at 450 nm. Cutoffs for C6 and VlsE ELISA were generated by dividing the absorbance of the low positive control by a fixed correction factor (2.60 for C6 ELISA or 3.43 for VlsE ELISA). Interpretation was based on the ratio of absorbance to cutoff or index value. Index values <0.9 were interpreted as negative, 0.9 to 1.1 as equivocal, and >1.1 as positive. C6 and VlsE ELISAs were validated in accordance with CLIA regulations.

### STTT and MTTT testing

Archival standard two-tier testing (STTT) results were used where available from the serum sources; for the remainder, STTT was carried out using the FDA-approved Captia *B. burgdorferi* IgG/IgM ELISA kit from Trinity and the FDA-cleared *B. burgdorferi* B31 IgG and *B. burgdorferi* B31 IgM immunoblot kits from Gold Standard Diagnostics. Modified two-tier testing (MTTT) was carried out on a subset of samples comprising 50 sera from Lyme disease patients with acute EM, 57 sera from patients with other conditions, and 54 of the 564 blood donor sera (49 non-endemic, five endemic) as controls. The Zeus Scientific Borrelia VlsE1/pepC10 IgG/IgM Test System was used as the first step, followed by separate Zeus Scientific *B. burgdorferi* IgG and IgM Test Systems, each a whole-cell sonicate ELISA, as the second step. These ELISAs were cleared by the FDA for use in combination in an MTTT protocol. The manufacturer’s criteria were used to interpret all test results.

The sensitivity and specificity of the Hybrid Lyme ELISA, used as a single-tier test, were compared with that of two-tier testing using STTT and MTTT methods, as well as with that of individual ELISAs based on either the C6 peptide or the recombinant VlsE protein in a conventional indirect ELISA format.

### Statistical analysis

ROC analysis was carried out using GraphPad Prism. 95% confidence intervals were calculated using the Exact Method.

## RESULTS

For analyses of sensitivity and specificity of the Hybrid Lyme ELISA, equivocal (indeterminate) results were regarded as positive, consistent with the interpretive criteria used for the MTTT. Four of the 101 Lyme disease patient sera (4.0%), but none of the 621 control sera that were tested on the Hybrid Lyme ELISA, yielded a result in the “equivocal” range (0.9 < index value < 1.1) .

### Comparison with the C6 ELISA and the VlsE ELISA

The Hybrid Lyme ELISA was compared with individual C6 and VlsE ELISAs using a set of 76 Lyme disease patient sera that were STTT-positive, including 32 with EM, and 100 blood donor sera from a non-endemic area as controls. The Hybrid Lyme ELISA demonstrated 97.4% sensitivity and 100% specificity, compared to 96.1% sensitivity and 98% specificity for the C6 ELISA, and 96.1% sensitivity and 95% specificity for the VlsE ELISA (Fig. 3). While the differences between the three assays’ results did not achieve statistical significance, the combination of sensitivity and specificity offered by the Hybrid Lyme ELISA could not be achieved by either the C6 ELISA or the VlsE ELISA merely by changing the cutoff values. Using the same ROC data as shown in Fig. 3, when the cutoffs of the C6 ELISA, the VlsE ELISA, and the Hybrid ELISA were each modified to yield either 99% specificity or 97.4% sensitivity, the corresponding sensitivity and specificity were higher by several percentage points for the Hybrid Lyme ELISA (Table 2). Furthermore, both the likelihood ratios and Matthews correlation coefficient, a combined measure of sensitivity, specificity, and positive and negative predictive value (19), indicated superior performance of the Hybrid Lyme ELISA over the other two assays (Fig. 3).

**TABLE 2:** Comparative sensitivity and specificity of ELISAs at equivalent specificity or sensitivity as calculated from the same ROC data used in Fig. 3

	Sensitivity		
All assays at specificity = 99.0%	Hybrid ELISA	VlsE ELISA	C6 ELISA
	97.4%	93.4%	94.7%

	Specificity		
All assays at sensitivity = 97.4%	Hybrid ELISA	VlsE ELISA	C6 ELISA
	100.0%	90.0%	97.0%

### Evaluation of CDC R67 panel

In an evaluation of the R67 Research Panel from CDC, the Hybrid Lyme ELISA detected 10/12 Lyme disease patient sera, while 8/12 were detected by STTT (Table 3). The two sera detected by the Hybrid Lyme ELISA that were missed by STTT were both from acute EM patients. Two other acute EM sera that tested negative by both the Hybrid Lyme ELISA and by STTT were also negative by whole-cell sonicate ELISA, the C6 ELISA, and immunoblots.

**TABLE 3:** Evaluation of CDC Research Panel R67 on Hybrid Lyme ELISA vs. standard two-tier testing^*a*^

	Hybrid ELISA Pos	Hybrid ELISA Neg	Two-Tier Pos	Two-Tier Neg	Total
Clinically Pos	10	2	8	4	12
Clinically Neg	0	20	0	20	20
Total	10	22	8	24	32

^*a*^ Lyme samples included four acute early Lyme (EM), four convalescent EM, two neurologic Lyme, and two Lyme arthritis. Two acute early Lyme (EM) samples were detected by Hybrid ELISA that were negative by STTT. Negative controls included sera from four healthy non-endemic and four healthy endemic individuals, two fibromyalgia, two mononucleosis, two severe periodontitis, two rheumatoid arthritis, two syphilis, and two multiple sclerosis patients. STTT results were provided by CDC.

### Comparison with STTT and MTTT

The Hybrid Lyme ELISA proved significantly (*P* < 0.05) more sensitive than the STTT for acute phase EM sera, detecting 47/50 (94%) or 30% (15/50) more than the STTT, which only detected 32/50 (64%) (*P* < 0.0001) (Table 4). Furthermore, 28/29 (96.6%) sera from patients with a single EM were detected by the Hybrid Lyme ELISA compared to 18/29 (62.1%) by STTT, a significant difference (*P* < 0.05) of 34.5%. Among patients with multiple EM, the 17.6% difference in sensitivity between the Hybrid Lyme ELISA, which detected 17/17 (100%), and the STTT, which detected 14/17 (82.4%), was not significant. Among sera from STTT-positive patients, including extracutaneous Lyme disease cases, the sensitivity of the Hybrid Lyme ELISA was 95.7% (45/47). The Hybrid Lyme ELISA demonstrated a 99.7% concordance with the STTT among the 621 control sera, with no significant difference (*P* = 0.5) between the test results (Table 4). Six out of 500 serum samples (1.2%) obtained from blood donors from non-endemic regions and one out of 64 (1.6%) from endemic regions were found positive by both the Hybrid Lyme ELISA and the STTT, all with a positive IgG immunoblot. Whether potentially due to exposure to *B. burgdorferi* during travel or other causes is unknown. Only two control serum samples out of the 564 donors (0.35%) tested positive by the Hybrid Lyme ELISA but negative by the STTT (with one sample IgG immunoblot positive but ELISA negative, and the other ELISA indeterminate and immunoblot negative), an insignificant difference (*P* > 0.05), suggesting that the addition of a second-step test to the Hybrid Lyme ELISA would not improve its overall performance. Both the Hybrid Lyme ELISA and STTT detected 0/57 sera from patients with a variety of other disease conditions that could lead to cross-reactivity. Accordingly, the overall specificities across the entire 621-member control panel for the Hybrid Lyme ELISA and STTT were 98.6% and 98.9%, respectively. Cross-reactivity was also evaluated on serum samples from patients with two other tick-borne infections, *Borrelia miyamotoi* and *Babesia microti*. Four out of five *B. miyamotoi* sera tested positive on the Hybrid Lyme ELISA, presumably due to the strong homology between the C6 peptide sequence of *B. burgdorferi* and that of the related *B. miyamotoi* (20, 21); however, prior or co-infection with *B. burgdorferi* cannot be ruled out, as three out of the four reactive samples also tested positive by the Lyme IgM immunoblot, which has been reported to be infrequently positive with *B. miyamotoi* sera (21).

**TABLE 4:** Comparison of detection rates by Hybrid Lyme ELISA and STTT in sera from Lyme disease patients, endemic and non-endemic blood donors, and individuals with other disease conditions^*a*^

Sample category	*n*	Hybrid ELISA			STTT			*p*
		# Positive	% Positive	95% CI	# Positive	% Positive	95% CI	
Early acute Lyme (EM)	50	47	94	83.5–98.8	32	64	49.2–77.1	6.10E-05
Single EM	29	28	96.6	82.2–99.9	18	62.1	42.3–79.3	0.002
Multiple EM	17	17	100	83.8–100	14	82.4	56.6–96.2	0.25
Extracutaneous/STTT-positive Lyme	47	45	95.7	85.5–99.5	47	100	93.8–100	0.5
Endemic blood donors	64	1	1.6	0.04–8.4	1	1.6	0.04–8.4	1
Non-endemic blood donors	500	8	1.6	0.7–3.1	6	1.2	0.4–2.6	0.5
Syphilis	7	0	0	–^*b*^	0	0	–	–
Influenza	4	0	0	–	0	0	–	–
*Helicobacter pylori*	5	0	0	–	0	0	–	–
CMV	5	0	0	–	0	0	–	–
EBV	5	0	0	–	0	0	–	–
RA	6	0	0	–	0	0	–	–
MS	7	0	0	–	0	0	–	–
HIV-1/2	8	0	0	–	0	0	–	–
COVID-19	6	0	0	–	0	0	–	–
Fibromyalgia	2	0	0	–	0	0	–	–
Periodontitis	2	0	0	–	0	0	–	–
Total other conditions	57	0	0	0–5.1	0	0	0–5.1	1
Total blood donors	564	9	1.6	0.7–3.0	7	1.2	0.5–2.5	0.5
Total controls	621	9	1.4	0.7–2.7	7	1.1	0.5–2.3	0.5

^*a*^ Early acute Lyme EM comprised sera that were both seropositive and seronegative. Within this group, single or multiple EM sera, where known, are listed separately. Extracutaneous/STTT-positive comprised sera that were STTT-positive, 12 of which were from patients with known extracutaneous manifestations of Lyme disease. The significance (p) of the difference in sensitivity and specificity between Hybrid ELISA and STTT was calculated by McNemar’s Test (two-tailed). Abbreviations: CMV, cytomegalovirus; EBV, Epstein-Barr virus; RA, rheumatoid arthritis; MS, multiple sclerosis.

^*b*^ "–”, indicates value not calculated.

One out of 7 *B. microti* sera was found equivocal on the Hybrid Lyme ELISA and was also reactive on a whole-cell sonicate Lyme disease ELISA and the C6 and VlsE ELISAs. False-positive Lyme disease testing in patients with babesiosis has been previously reported, though not with the C6 peptide (11), and Lyme disease and babesiosis coinfections are known to occur.

In a comparison of the Hybrid Lyme ELISA with the MTTT, we tested the same set of 50 EM patient sera and 57 sera from patients with other disease conditions that were previously tested with the STTT (see Table 4), supplemented by 54 sera from non-endemic blood donors. The Hybrid Lyme ELISA was 18% higher in sensitivity than the MTTT in detection of early acute EM sera (47/50 or 94% vs. 38/50 or 76%, respectively), a significant (*P* < 0.05) difference (Table 5). Notably, nine out of the 12 acute EM sera that were negative by MTTT were detected by the Hybrid Lyme ELISA, while the three acute EM sera that were negative by the Hybrid Lyme ELISA were also negative by the MTTT and by both steps of the STTT carried out separately. Among sera from patients with a single EM, the Hybrid Lyme ELISA detected 27.6% (8/29) more sera than the MTTT, yielding sensitivities of 96.6% (28/29) vs. 69% (20/29), respectively, a significant difference (*P* < 0.05). By comparison, both the Hybrid Lyme ELISA and the MTTT detected 17/17 (100%) of sera from patients with multiple EM skin lesions. The Hybrid Lyme ELISA was nominally higher but statistically equivalent in specificity to the MTTT (*P* = 0.5). The difference of 0.198 in Youden index for the two assays further reflects the superior overall performance of the Hybrid Lyme ELISA. As this study compared the Hybrid Lyme ELISA with MTTT ELISAs from a single manufacturer, how broadly these findings may apply to other MTTT assays is not clear. However, a recent study reported positive and negative percent agreement of ≥95% for three currently available commercial FDA-approved MTTT assays compared to the STTT, suggesting that these three MTTT assays have similar sensitivity and specificity (12).

**TABLE 5:** Sensitivity and specificity of Hybrid ELISA vs. modified two-tier testing (MTTT)^*a*^

Group	Sample category	*n*	Hybrid ELISA positive			MTTT positive			*p*
			*n*	%	95% CI	*n*	%	95% CI	
A	Early acute Lyme (EM)	50	47	94.0	83.5–98.8	38	76.0	61.8–86.9	0.004
	Single EM	29	28	96.6	82.2–99.9	20	69.0	49.2–84.7	0.008
	Multiple EM	17	17	100.0	83.8–100	17	100.0	83.8–100	1.0
B	Other disease conditions	57	0	0.0	0–5.1	1	1.8	0.04–9.4	1.0
C	Blood donors	54	0	0.0	0–5.4	1	1.9	0.05–9.9	1.0
D	Healthy control and other disease conditions	111	0	0.0	0–2.7	2	1.8	0.2–6.4	0.5
	Youden Index (A–D)	NA^*b*^	NA	0.940	NA	NA	0.742	NA	NA

^*a*^ Early Acute Lyme (EM) comprised sera that were both seropositive and seronegative. Within this group, single or multiple EM sera, where known, are listed separately. MTTT testing was carried out using FDA-approved ELISA kits from Zeus Scientific, with a VlsE/pepC10 ELISA as 1st step and IgG and IgM whole-cell sonicate-based ELISAs as 2nd step. The MTTT result was interpreted as positive if the 1st step and either IgG or IgM 2nd step tests were positive or indeterminate, according to the package insert. Youden index (J statistic) was calculated as (sensitivity + specificity – 1). The significance (*P*) of the difference in sensitivity and specificity between Hybrid ELISA and MTTT was calculated by McNemar’s Test (two-tailed).

^*b*^ NA, not applicable.

### Hybrid Lyme ELISA sensitivity vs. time after onset of disease

**Fig 4:**
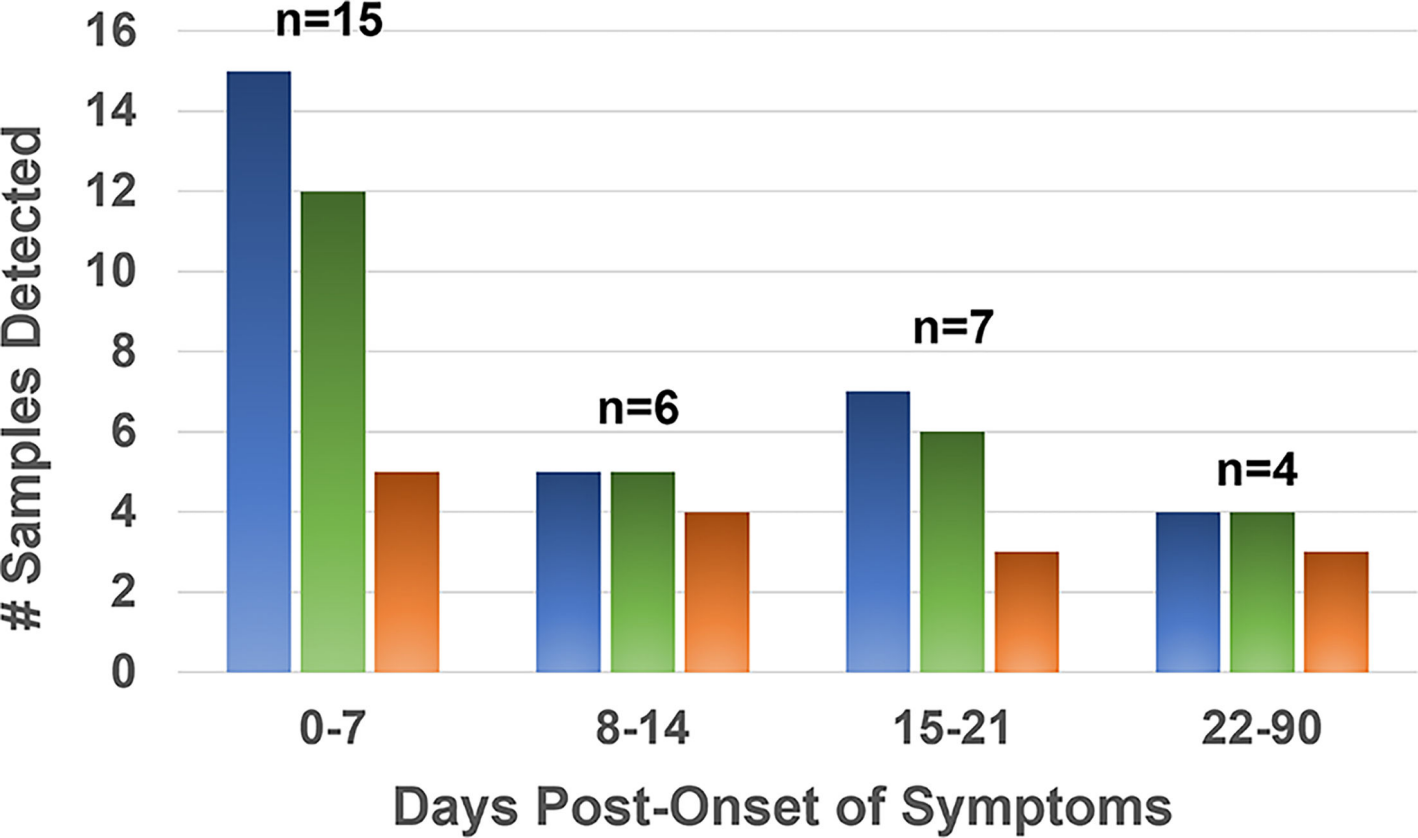
Detection of EM (early Lyme disease) sera vs. days after onset of symptoms. Hybrid Lyme ELISA (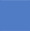), MTTT (), and STTT ().

With respect to the sensitivity of detection of each method relative to the time after onset of symptoms, we tested a subset of 32 serum samples from early Lyme disease patients presenting with EM, for whom the dates of onset of symptoms and sample draw were known. The Hybrid Lyme ELISA proved significantly more sensitive than STTT for detection of samples collected within seven days of onset, detecting 15/15 serum samples compared to 5/15 by STTT (*P* < 0.05) (Fig. 4). While the Hybrid Lyme ELISA was likewise more sensitive than STTT for detection of samples collected at later time points, these differences did not reach significance given the small sample sizes; however, the overall difference across all time points (31/32 vs. 15/32) was significant (*P* < 0.0001). The Hybrid Lyme ELISA was also more sensitive than the MTTT (15/15 vs. 12/15) at 0–7 days post onset, although the difference was not significant.

### Reproducibility

Reproducibility of the Hybrid Lyme ELISA was assessed using a panel of eight sera from Lyme disease patients with absorbance values ranging from 0.041 to 0.661, of which six sera had absorbances < 0.120, and seven sera from blood donors with absorbance values ranging from 0.042 to 0.069. All samples were tested in three independent assay runs performed by three different operators. The average coefficient of variation (CV) of the ELISA absorbance for the three assay runs among the 13 samples with average absorbance <0.12 was 8.8%, with a standard deviation of 4.7%. Among the Lyme disease patient and the blood donor groups, the average CVs were 7.1% and 10.3%, respectively. No discordance in interpretation was observed for any of the positive or negative control samples across the three independent assay runs, based on the index value calculated as described in the Materials and Methods. Based on CV values, the Hybrid Lyme ELISA was thus found to perform with a high level of reproducibility, including for samples with low absorbance values around the cutoff.

## DISCUSSION

The STTT algorithm adopted in 1994 has been relatively successful at bringing high specificity to Lyme disease testing, but at the cost of low sensitivity in early Lyme disease manifesting as the EM skin lesion (6). With the recent advent of MTTT, new diagnostic options are available, but the second step test for either of these algorithms continues to add cost, operational complexity, and additional time needed for completion of the testing process.

The Hybrid Lyme ELISA makes use of the C6 peptide and the VlsE protein from which it is derived, each of which has been shown in multiple studies to offer both high sensitivity and high specificity in detection of antibodies elicited by various pathogenic *Borrelia* species that cause Lyme disease (9, 10, 13, 22–32). The design of this ELISA originated from the observation in earlier studies that serum samples from controls exhibiting false reactivity with the C6 peptide rarely exhibited similar false reactivity with the recombinant VlsE protein, and vice-versa (9) (and unpublished data). This suggested that the epitopes responsible for such artifactual reactivity were not shared between the two sequences. We postulated the inverse, that epitopes that were shared between C6 peptide and VlsE protein would prove to be specific for Lyme disease. Accordingly, the Hybrid Lyme ELISA was designed to detect only the subset of antibodies in Lyme disease patient sera that recognize both a linear epitope in the isolated C6 peptide and the homologous epitope within the VlsE protein. The Hybrid Lyme ELISA is based on the principle that an individual multivalent antibody can bind the C6 peptide at one binding site and the recombinant VlsE protein at another in a bridge format, where one antigen is immobilized on the solid phase and the other is conjugated to an enzymatic reporter (Fig. 2). Antibodies that bind solely to one of the two antigens are not detected. This principle appears to lead to a higher binding stringency, resulting in negligible spurious or false-positive reactivity compared with assays using traditional second antibody–enzyme conjugates. These characteristics allow for a relatively low cutoff value below the range of conventional indirect ELISAs, without the risk of non-specific reactivity. This may enable the detection of the relatively low antibody titers present in patients at early time periods after onset of infection, which would otherwise not rise above the level of non-specific background reactivity. The average cutoff value for the Hybrid Lyme ELISA was 0.081 absorbance units, compared with 0.429 for the STTT ELISA and 0.457 for the MTTT ELISAs, both over five-fold higher. This feature may explain how the Hybrid Lyme ELISA’s sensitivity could exceed that of other Lyme ELISAs, particularly enabling the detection of EM at early time points after infection. Supporting this interpretation, 9/15 acute EM sera that were detected by the Hybrid Lyme ELISA but were negative by STTT—and similarly 4/5 acute EM sera that were detected by the Hybrid Lyme ELISA but were negative by MTTT—had absorbance values <0.2 in the Hybrid Lyme ELISA. Detection of these sera contributed to the Hybrid Lyme ELISA’s sensitivity advantage of 30% over STTT and 18% over MTTT in sera from EM patients—and more significantly, a 35% advantage over STTT and 28% over MTTT in patients with a single EM skin lesion.

Many conventional serologic assays use an indirect ELISA format in which serum antibodies are detected by an enzyme–antibody conjugate, offering the advantages that commercial off-the-shelf reagents can be used for the detection step and that specificity for a given antibody class (e.g., IgG or IgM) and host species can be built into the assay. However, these advantages are offset by a significant disadvantage—the non-specific binding inherent to antibody conjugates, which leads to a level of non-specific reactivity and false positivity (often 1% or more [33–35]), which degrades assay performance. In the context of Lyme disease, where more than 3 million tests are performed annually in the United States, a 1% false-positive rate would potentially translate to 30,000 false-positive diagnoses (36). In addition to the unnecessary prescription of antibiotics, such false-positive diagnoses may divert attention away from the true cause of the patient’s illness and delay the appropriate needed treatment.

An advantage of the Hybrid Lyme ELISA format is that a secondary antibody conjugate is not necessary for serum antibody detection, thereby eliminating the associated non-specific reactivity. Furthermore, the assay can be reduced to a near-homogeneous format, as all components other than the enzyme substrate can be added simultaneously. As such, it is simpler and faster than most conventional ELISA protocols. Secondly, with low non-specific binding, serum can be assayed at low dilution or even neat, which allows for higher analytical sensitivity. Additionally, the Hybrid Lyme ELISA allows antibody binding and detection independent of antibody class or host species. This may be an advantage where it is desirable to detect antibodies of all classes, e.g., IgG and IgM, or to use the same assay for humans and non-human reservoir species.

In this study, we demonstrated the feasibility of a novel assay termed the Hybrid Lyme ELISA, providing evidence that its specificity was comparable to both the STTT and MTTT protocols, while its sensitivity for detection of early Lyme disease (EM) was significantly better than that of the STTT (*P* < 0.0001) and also the MTTT (*P* = 0.004) protocols that were evaluated. In particular, the Hybrid Lyme ELISA was significantly more sensitive than both the STTT (*P* = 0.002) and MTTT (*P* = 0.008) for detecting Lyme borrelial antibody in patients with a single EM, arguably the earliest stage with respect to timing and the most challenging manifestation of Lyme disease to diagnose using serologic testing. The observed sensitivity of over 90% makes this assay especially relevant for Lyme disease patients with EM and, in this regard, distinguishes it from both the STTT and MTTT assays in a very desirable way.

A limitation of the Hybrid Lyme ELISA is that it does not distinguish between IgM and IgG reactivity. This may be a shortcoming where there is a need to confirm a diagnosis of early- versus late-stage Lyme disease. In cases where this is important, however, IgG- and IgM-specific second-tier testing could follow the Hybrid Lyme ELISA. Another limitation of the Hybrid Lyme ELISA in regard to specificity for diagnosing Lyme disease is that a false-positive test might occur as a result of *B. miyamotoi* infection, and this should be further investigated. One consequence of cross-reactivity is the potential for some degree of overreporting of Lyme disease cases when surveillance is based on laboratory testing results alone. Additionally, patients without active, symptomatic Lyme disease may still yield positive results on this and other immunoassays due to prior infection with *B. burgdorferi*. The rate of background seropositivity for *B. burgdorferi* in endemic populations in the United States has been reported to range from 1.4% to 9.4% in individual assays, depending on the populations studied (10, 37, 38). It should also be noted that two serum samples from Lyme disease patients without an EM skin lesion that were positive by STTT (but were not tested by MTTT) were not detected by the Hybrid Lyme ELISA. One serum sample was from a patient with carditis and the other was from a patient with flu-like symptoms, and interestingly, both patients tested IgG-negative but IgM-positive by immunoblot. While not a significant difference, the basis for this lack of detection nevertheless merits further investigation.

Limitations of this study include the use of previously collected, well-characterized serum samples from Lyme disease patients and the use of blood donor sera as negative controls. Serum samples were not blinded prior to performing the assays; however, interpretation of results was done by automated calculations rather than manually. The number of Lyme disease serum samples tested was relatively small, and expanding this number, especially EM patient sera, will be important to validate the observations in this study and to refine the Hybrid Lyme ELISA’s performance metrics. Furthermore, because the majority of EM sera in this study were positive by at least a first-tier test, and two-tier seropositivity was a requisite criterion for definition of Lyme disease in the absence of EM, the sensitivity of any serologic assay is likely to be higher in these groups. Additionally, the performance of the Hybrid Lyme ELISA was compared against only one version of the STTT and MTTT assay kits, and other kits might differ in performance. Prospective clinical studies on patients with suspected tick-borne disease are now underway to evaluate the performance of the Hybrid Lyme ELISA as a one-tier test for diagnosing Lyme disease, in comparison with MTTT and STTT testing.

In conclusion, in this study, the single-tier Hybrid Lyme ELISA was shown to provide greater sensitivity for EM and equivalent specificity in comparison to both STTT and MTTT testing. This ELISA may thus facilitate the transformation of routine testing for Lyme disease from a two-step to a one-step testing algorithm, with the potential to be used to diagnose patients at all stages of the disease.
